# Lats2 promotes heart failure by stimulating p53-mediated apoptosis during pressure overload

**DOI:** 10.1038/s41598-021-02846-3

**Published:** 2021-12-06

**Authors:** Dan Shao, Peiyong Zhai, Chengchen Hu, Risa Mukai, Sebastiano Sciarretta, Dominic Del Re, Junichi Sadoshima

**Affiliations:** 1 Department of Cell Biology and Molecular Medicine, Rutgers New Jersey Medical School, 185 S Orange Ave, MSB G609, Newark, NJ 07103 USA; 2 Department of Medical and Surgical Sciences and Biotechnologies, Sapienza University of Rome, Latina, Italy; 3 IRCCS Neuromed, Pozzilli, IS Italy

**Keywords:** Cardiology, Molecular medicine

## Abstract

The Hippo pathway plays a wide variety of roles in response to stress in the heart. Lats2, a component of the Hippo pathway, is phosphorylated by Mst1/2 and, in turn, phosphorylates YAP, causing inactivation of YAP. Lats2 stimulates apoptosis and negatively affects hypertrophy in cardiomyocytes. However, the role of Lats2 during cardiac stress is poorly understood in vivo. Lats2 is activated in the mouse heart in response to transverse aortic constriction (TAC). We used systemic Lats2 +/- mice to elucidate the role of endogenous Lats2. Cardiac hypertrophy and dysfunction induced by 4 weeks of TAC were attenuated in Lats2 +/- mice, and interstitial fibrosis and apoptosis were suppressed. Although TAC upregulated the Bcl-2 family proapoptotic (Bax and Bak) and anti-apoptotic (Bcl-2 and Bcl-xL) molecules in non-transgenic mice, TAC-induced upregulation of Bax and Bak was alleviated and that of Bcl-2 was enhanced in Lats2 +/- mice. TAC upregulated p53, but this upregulation was abolished in Lats2 +/- mice. Lats2-induced increases in apoptosis and decreases in survival in cardiomyocytes were inhibited by Pifithrin-α, a p53 inhibitor, suggesting that Lats2 stimulates apoptosis via a p53-dependent mechanism. In summary, Lats2 is activated by pressure overload, thereby promoting heart failure by stimulating p53-dependent mechanisms of cell death.

## Introduction

Heart failure remains the number one cause of death in developed countries and the number of patients is increasing, despite the remarkable progress in medical treatment, including effective coronary interventions for acute myocardial infarction patients^1^. Various mechanisms facilitate the progression of heart failure. Among these, cardiomyocyte death reduces the number of functional cardiomyocytes in the adult heart, which in turn plays an essential role in mediating the development of heart failure^2^.

The Hippo pathway is an evolutionarily conserved signaling mechanism that plays an important role in regulating organ size and tumorigenesis^3^. The hippo pathway includes upstream kinases, including Mst1, Mst2, Lats1 and Lats2, and downstream nuclear transcription co-factors, including YAP and TAZ. Mst1 and Mst2 activated by environmental stress and other stimuli induce phosphorylation of Lats1 and Lats2, which in turn induce phosphorylation of YAP and TAZ. When YAP is phosphorylated at Ser127, it exits the nucleus and becomes inactivated. On the other hand, when YAP is unphosphorylated at Ser127, it is localized in the nucleus and stimulates the activity of transcription factors, including TEAD1, which in turn activates cell proliferation and cell survival^4,5^.

We have shown previously that Mst1 is activated by cardiac stress, including ischemia/reperfusion in the heart, inducing apoptosis and suppressing autophagy in cardiomyocytes^6–8^. Lats2 is also activated by cardiac stress and stimulation of Mst1, in turn stimulating apoptosis and inhibiting cardiac hypertrophy^9,10^. On the other hand, YAP mediates cardiac hypertrophy, cardiomyocyte cell cycle reentry and cell survival, mediated through interaction with TEAD and FoxO transcription factors^5,10–14^.

We have shown previously that Lats2 plays an important role in mediating apoptosis and inhibiting hypertrophy in cardiomyocytes in vitro^9^. Interestingly, cardiac-specific overexpression of dominant-negative Lats2 inhibits cardiomyocyte apoptosis and enhances cardiac hypertrophy in mice in response to pressure overload^9^. Nevertheless, how inhibition of Lats2 affects cardiac function during chronic pressure overload remains unclear. In addition, dominant negative Lats2 inhibits both Lats1 and Lats2^9^ and protein overexpression could elicit unphysiological effects in the heart.

Thus, the goals in this study were to examine the effects of a loss of Lats2 function, using systemic Lats2 heterozygous KO (Lats2 +/-) mice, and investigate the role of endogenous Lats2 in cardiac hypertrophy and heart failure during pressure overload.

## Results

### Systemic Lats2 heterozygous downregulation only mildly affects the heart at baseline

**Figure 1:**
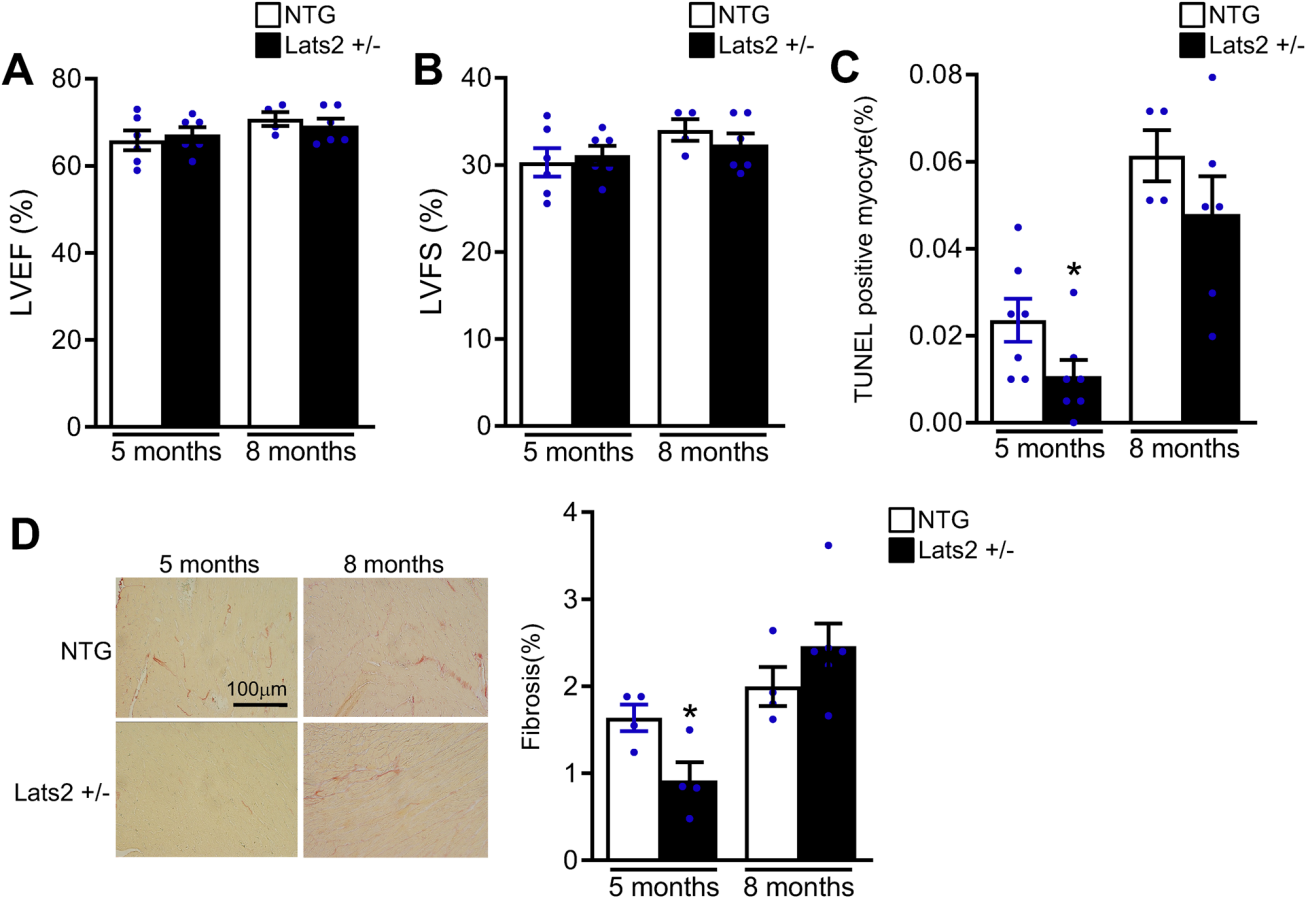
Baseline phenotype of Lats2 +/- mouse hearts. Non-transgenic (NTG) and Lats2 +/- mice were euthanized at 5 or 8 months of age. (**A**) and (**B**), Left ventricular ejection fraction (LVEF) (**A**) and left ventricular fractional shortening (LVFS) (**B**) were evaluated with echocardiographic analyses. (**C**), Myocardial apoptosis was evaluated with TUNEL assays. (**D**), Myocardial fibrosis was evaluated with Picrosirius Red staining. *p < 0.05 versus sham hearts.

In order to investigate the role of endogenous Lats2, we studied the effects of a loss of Lats2 function. We used systemic heterozygous Lats2 KO (Lats2 +/-) mice because homozygous genetic deletion of Lats2 is embryonic lethal. Left ventricular (LV) weight/body weight (LVW/BW) at baseline did not differ significantly between Lats2 +/- and non-transgenic (NTG) mice at 5 months of age. LVW/BW was significantly greater in Lats2 +/- mice at 8 months of age than in NTG mice (Supplemental Tables S1 and S2). At both 5 and 8 months of age, the cardiac dimension and LV function in Lats2 +/- mice, evaluated with echocardiographic and hemodynamic measurements, were not significantly different from those in NTG mice except that the septal wall was slightly thinner in Lats2 +/- mice than in NTG mice at 8 months of age (Fig. 1A,B, Supplemental Tables S3 and S4). Histological analyses showed that there were significantly fewer TUNEL positive cardiomyocytes and significantly less cardiac fibrosis in Lats2 +/- mice than in NTG mice at 5 months of age, but not at 8 months of age (Fig. 1C,D). Taken together, these observations suggest that endogenous Lats2 may slightly promote apoptosis and cardiac fibrosis at 5 months of age but does not significantly affect LV function at baseline. The reason why the mild decrease in both fibrosis and apoptosis observed at 5 months of age was normalized at 8 months of age is unclear.

### Endogenous Lats2 promotes cardiac dysfunction in response to pressure overload

**Figure 2:**
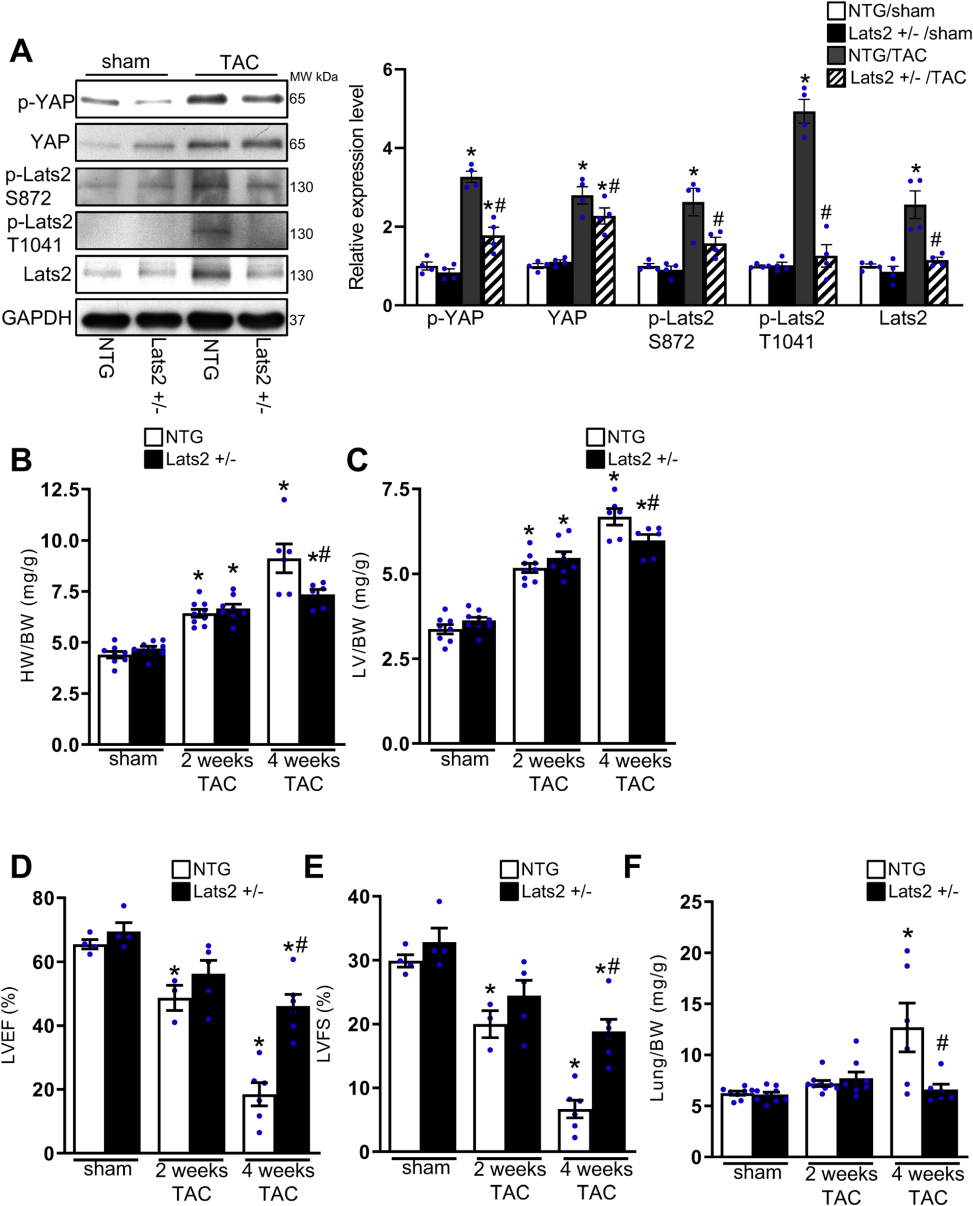
The effect of pressure overload in non-transgenic (NTG) and Lats2 +/- mice. NTG and Lats2 +/- mice were subjected to either transverse aortic constriction (TAC) or sham operation for either 2 or 4 weeks. The results from the 2 and 4 weeks sham groups were combined. (**A**), The levels of Ser127 phosphorylated YAP, total YAP, Ser872 phosphorylated Lats2, Thr1041 phosphorylated Lats2, total Lats2, and GAPDH in the heart homogenates prepared from NTG and Lats2 +/- mice subjected to either TAC or sham operation for 4 weeks. Representative images (left) and quantification (right) are shown (n = 4). (**B**) and (**C**), Heart weight/body weight (HW/BW) (**B**) and left ventricular weight/body weight (LVW/BW). (**C**) and (**D**), Left ventricular ejection fraction (LVEF) (**D**) and left ventricular fractional shortening (LVFS) (**E**) were evaluated with echocardiographic analyses. (**F**), Lung weight/body weight (LungW/BW). In (**A**–**F**), *p < 0.05 versus NTG mouse hearts with sham operation. #*p* < 0.05 versus NTG mouse heart with TAC for 4 weeks.

We next investigated the role of endogenous Lats2 during pressure overload. To this end, Lats2 +/- mice and NTG mice were subjected to TAC or sham operation for 2 and 4 weeks. The mice were subjected to echocardiographic analyses and hemodynamic analyses before euthanasia. Four weeks of TAC increased Ser872 and Thr1041 phosphorylation of Lats2^15^, suggesting that Lats2 is activated in the heart in response to TAC. Four weeks of TAC also increased YAP Ser127 phosphorylation, which was attenuated in Lats2 +/- mice. Lats2 directly phosphorylates YAP at Ser127^16^ (Fig. 2A). Thus, these results suggest that Lats2 plays a critical role in mediating Ser127 phosphorylation of YAP in the heart in response to TAC.

Heart weight/body weight (HW/BW) and left ventricular weight/body weight (LVW/BW) were significantly increased after 2 and 4 weeks of TAC. Although there was no significant difference in HW/BW or LV/BW after 2 weeks of TAC between Lats2 +/- and NTG mice, both ratios were significantly smaller in Lats2 +/- mice than in NTG mice after 4 weeks of TAC (Fig. 2B,C). These results suggest that endogenous Lats2 positively affects cardiac hypertrophy in response to 4 weeks of TAC. In NTG mice, LV ejection fraction (LVEF) and LV fractional shortening (LVFS), indexes of LV systolic function, evaluated via echocardiographic measurements, were significantly decreased 2 and 4 weeks after TAC compared to in mice that underwent sham operation. LVEF and LVFS were also decreased 4 weeks after TAC compared to in mice that underwent sham operation in Lats2 +/- mice, but their values were significantly higher in Lats2 +/- mice than in NTG mice (Fig. 2D,E). Hemodynamic measurements showed that LV systolic pressure was significantly lower in NTG mice than in Lats2 +/- mice, most likely due to more severe LV dysfunction in NTG mice than in Lats2 +/- mice 4 weeks after TAC. It is unlikely that the TAC applied to Lats2 +/- mice was more severe since the initial development of hypertrophy and LV dysfunction was similar between NTG and Lats2 +/- mice. Lung weight/body weight (LungW/BW), an index of lung congestion, was significantly elevated in NTG mice 4 weeks after TAC, consistent with the presence of heart failure. In contrast, LungW/BW was not elevated in Lats2 +/- mice 2 and 4 weeks after TAC compared to in response to sham operation. LungW/BW 4 weeks after TAC was significantly smaller in Lats2 +/- mice than in NTG mice (Fig. 2F). Taken together, these results suggest that pressure overload-induced cardiac hypertrophy, LV dysfunction and heart failure were significantly alleviated in Lats2 +/- mice compared to in NTG mice.

**Figure 3:**
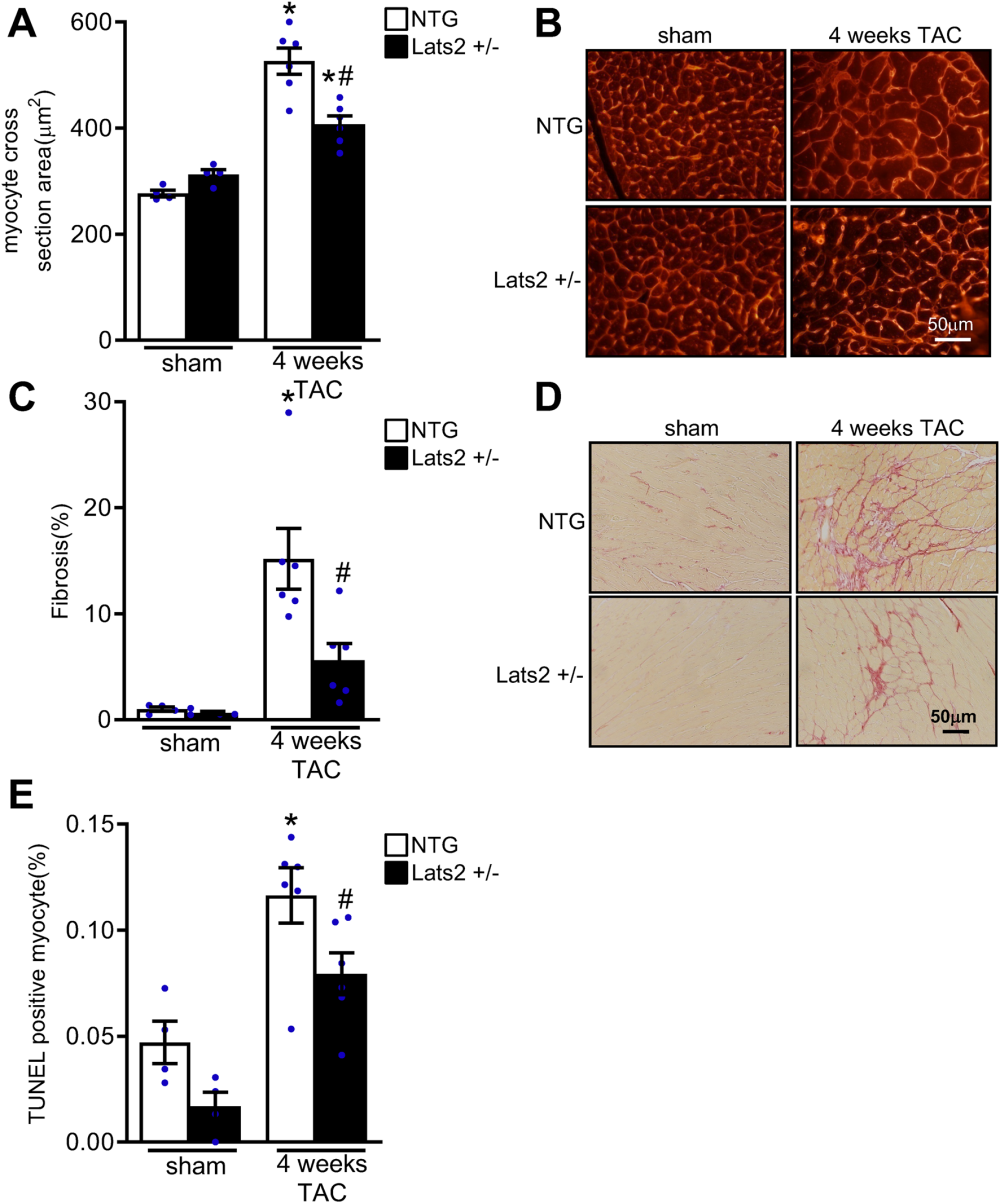
The effect of pressure overload upon cardiac hypertrophy, fibrosis and apoptosis in non-transgenic (NTG) and Lats2 +/- mice. NTG and Lats2 +/- mice were subjected to either transverse aortic constriction (TAC) or sham operation for 4 weeks. (**A**) and (**B**), The size of left ventricular cardiomyocytes was evaluated with wheat germ agglutinin staining and ImageJ analyses. Representative images (**A**) and quantification (**B**) are shown. (**C**) and (**D**), Interstitial fibrosis was evaluated with Picrosirius Red staining and ImageJ analyses. Representative images (**C**) and quantification (**D**) are shown. (**E**), Cardiomyocyte apoptosis was evaluated with TUNEL assays and ImageJ analyses. Quantification is shown. In (**A**), (**C**) and (**E**), *p < 0.05 versus NTG mouse hearts with sham operation. #*p* < 0.05 versus NTG mouse heart with TAC for 4 weeks.

Four weeks of TAC significantly increased histologically evaluated cardiomyocyte cross sectional area in both NTG and Lats2 +/- mice. However, the increase in cardiomyocyte cross sectional area was significantly smaller in Lats2 +/- mice than in NTG mice (Fig. 3A,B). Similarly, 4 weeks of TAC significantly increased interstitial fibrosis in the heart evaluated with Picro Sirius Red staining in both NTG and Lats2 +/- mice, but the increase in interstitial fibrosis was significantly smaller in Lats2 +/- mice than in NTG mice (Fig. 3C,D). Likewise, 4 weeks of TAC significantly increased apoptosis in the heart as evaluated with TUNEL staining, in both NTG and Lats2 +/- mice, but the increase was significantly smaller in Lats2 +/- mice than in NTG mice (Fig. 3E).

### Endogenous Lats2 positively regulates Bax and Bak in the heart in response to pressure overload

**Figure 4:**
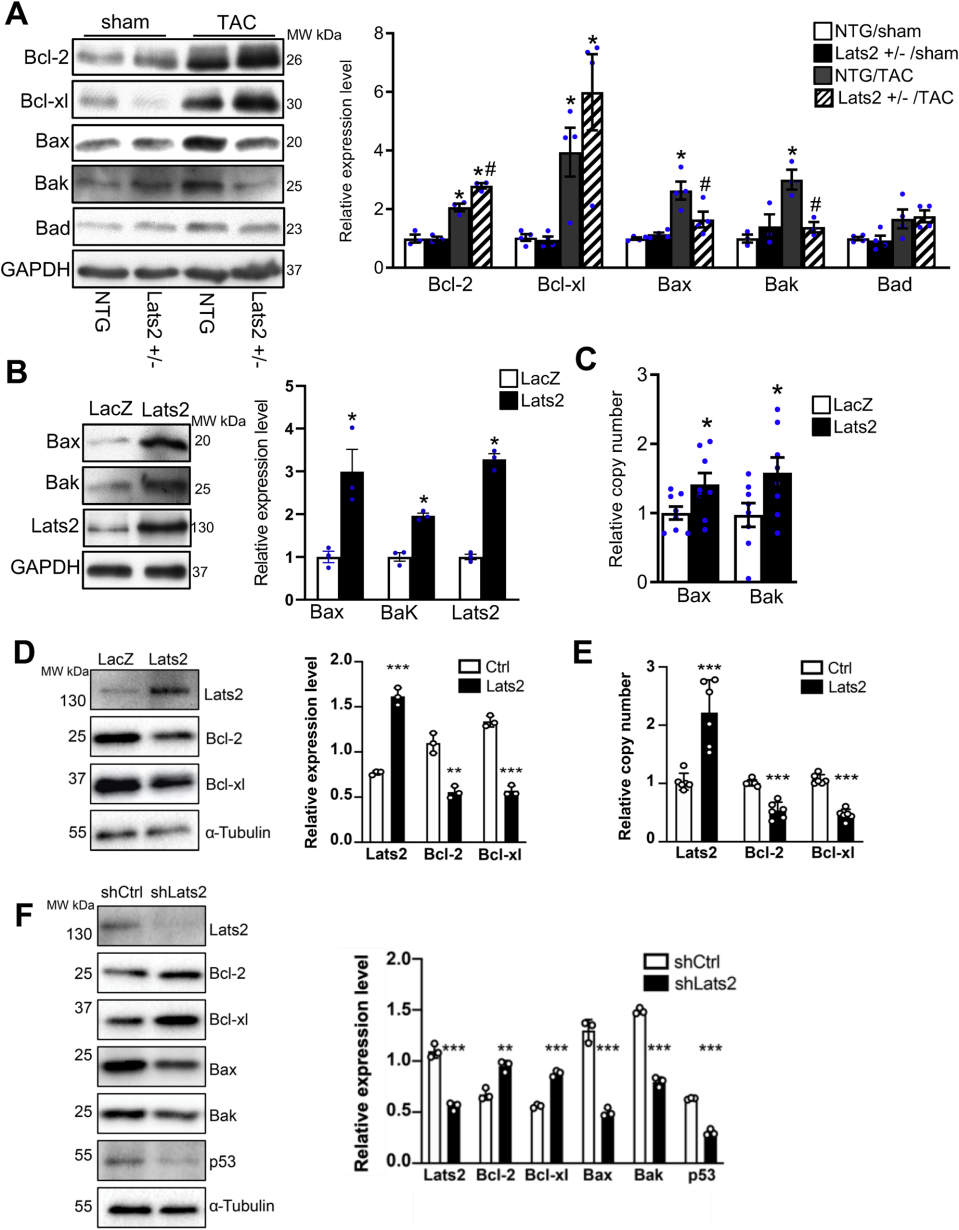
The role of Lats2 in mediating expression of Bcl-2 family proteins during pressure overload. (**A**), Non-transgenic (NTG) and Lats2 +/- mice were subjected to either transverse aortic constriction (TAC) or sham operation for 4 weeks. The levels of Bcl-2, Bcl-xL, Bax, Bak, Bad and GAPDH were evaluated with immunoblot analyses. Representative images (left) and quantification (right) are shown (n = 4). *p < 0.05 versus NTG mouse hearts with sham operation. #*p* < 0.05 versus NTG mouse heart with TAC for 4 weeks. (**B**–**E**), Cultured neonatal rat ventricular cardiomyocytes were transduced with adenovirus harboring LacZ or Lats2. B, The levels of Bax, Bak, Lats2 and GAPDH were evaluated with immunoblot analyses. Representative images (left) and quantification (right) are shown (n = 4). (**C**), mRNA levels of Bax and Bak were evaluated with qPCR analyses. *p < 0.05 versus Adenovirus harboring LacZ. (**D**), The levels of Bax, Bak, Lats2 and α-Tubulin were evaluated with immunoblot analyses. Representative images (left) and quantification (right) are shown. (n = 3). (**E**), mRNA levels of Bax and Bak were evaluated with qPCR analyses. (n = 6). *p < 0.05, **p < 0.01, ***p < 0.001 versus Adenovirus harboring LacZ. (**F**), Cultured neonatal rat ventricular cardiomyocytes were transduced with adenovirus harboring shRNA Lats2 or shRNA control. The levels of Lats2, Bcl-2, Bcl-xL, Bax, Bak, p53 and α-tubulin were evaluated with immunoblot analyses. Representative images (left) and quantification (right) are shown (n = 3). *p < 0.05, **p < 0.01, ***p < 0.001 versus Adenovirus harboring shRNA control.

Since Lats2 promotes apoptosis in cardiomyocytes^9^, we investigated how the haploinsufficiency of Lats2 affects expression of Bcl-2 family proapoptotic and prosurvival proteins. TAC upregulated protein expression of Bax and Bak, proapoptotic proteins, compared to sham operation in NTG mice whereas TAC-induced upregulation of Bax and Bak was significantly attenuated in Lats2 +/- mice. TAC did not significantly change the protein level of Bad, another proapoptotic protein, in either NTG or Lats2 +/- mice compared to sham operation (Fig. 4A). TAC also upregulated Bcl-2 and Bcl-xL, anti-apoptotic proteins, compared to sham operation in both NTG and Lats2 +/- mice. However, TAC-induced upregulation of Bcl-2 was significantly greater in Lats2 +/- mice than in NTG mice and that of Bcl-xL also tended to be greater in Lats2 +/- mice than in NTG mice (Fig. 4A). Adenovirus-mediated transduction of Lats2 induced significant upregulation of Bax and Bak proteins and mRNAs compared to adenovirus-mediated transduction of LacZ (control) in cultured neonatal rat ventricular cardiomyocytes (Fig. 4B,C), suggesting that Lats2-induced upregulation of Bax and Bak in cardiomyocytes is cell-autonomous. Overexpression of Lats2 significantly downregulated Bcl-2 and Bcl-xL protein and mRNA expression in cultured neonatal rat ventricular cardiomyocytes (Fig. 4D,E), suggesting that Lats2 downregulates anti-apoptotic proteins in the Bcl-2 family in a cell autonomous manner. We also conducted Lats2 loss-of-function experiments in cultured neonatal rat ventricular cardiomyocytes. shRNA-mediated downregulation of Lats2 induced significant downregulation of Bax and Bak and upregulation of Bcl-2 and Bcl-xL compared to control shRNA treatment (Fig. 4F), consistent with the in vivo result. Thus, the effect of loss of Lats2 function upon Bcl-2/Bcl-xL is also cell autonomous in cardiomyocytes.

**Figure 5:**
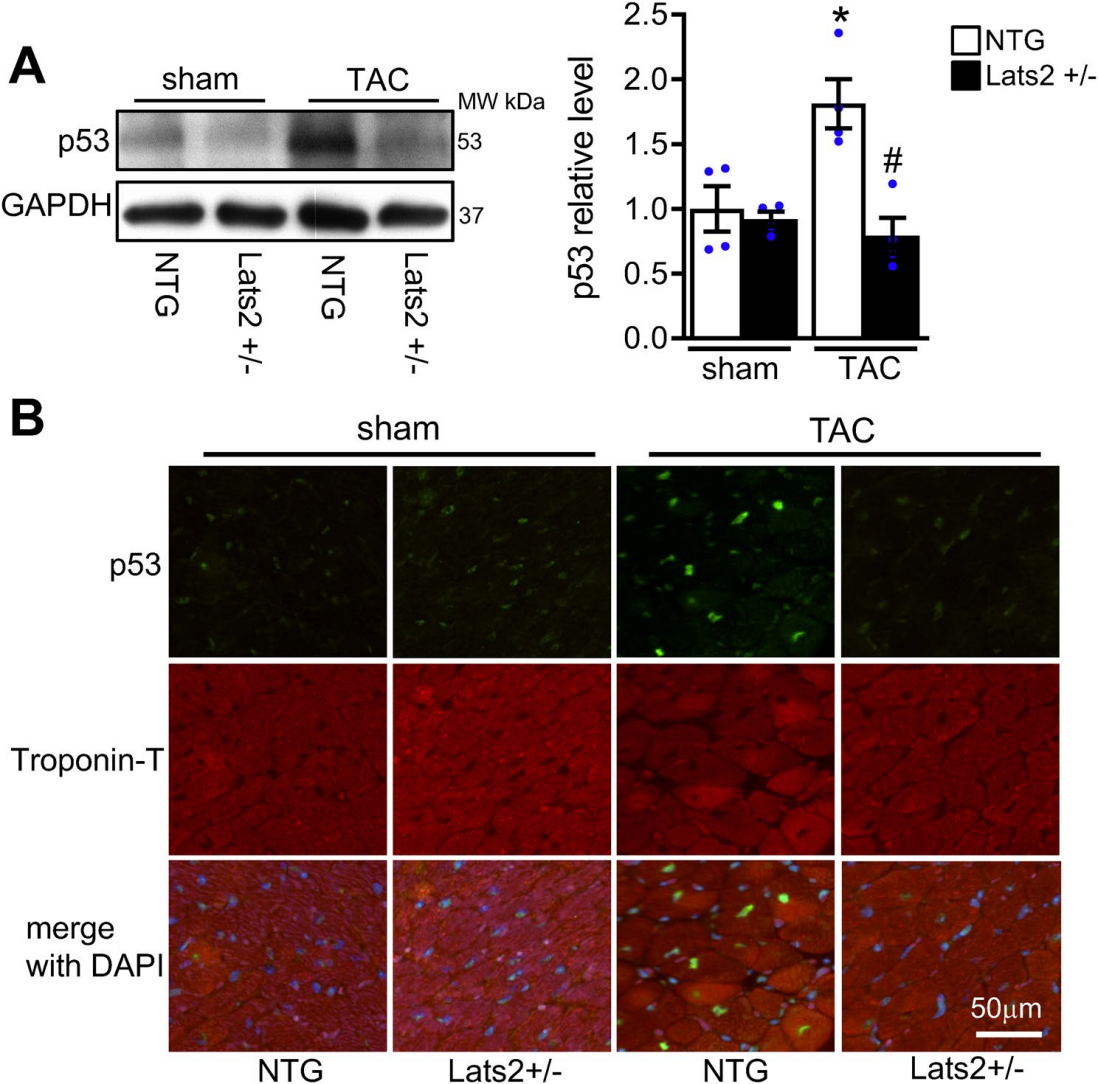
The role of Lats2 in mediating p53 in the heart during pressure overload. Non-transgenic (NTG) and Lats2 +/- mice were subjected to either transverse aortic constriction (TAC) or sham operation for 4 weeks. (**A**), The levels of p53 and GAPDH were evaluated with immunoblot analyses. Representative images (left) and quantification (right) are shown. *p < 0.05 versus NTG mouse hearts with sham operation. #*p* < 0.05 versus NTG mouse hearts with TAC for 4 weeks. (**B**), The level of p53 in the heart was evaluated by immunostaining with anti-p53 antibody and anti-troponin-T antibody, the latter of which identifies cardiomyocytes. Representative images are shown.

Immunoblot analyses showed that 4 weeks of TAC upregulates p53 in NTG mouse hearts, an effect that was abolished in the Lats2 +/- mouse heart (Fig. 5A). The results were confirmed with immunostaining of myocardial sections. The TAC-induced upregulation of p53 nuclear staining in Troponin-T positive cells, namely cardiomyocytes, observed in NTG mice was inhibited in Lats2 +/- mice (Fig. 5B).

**Figure 6:**
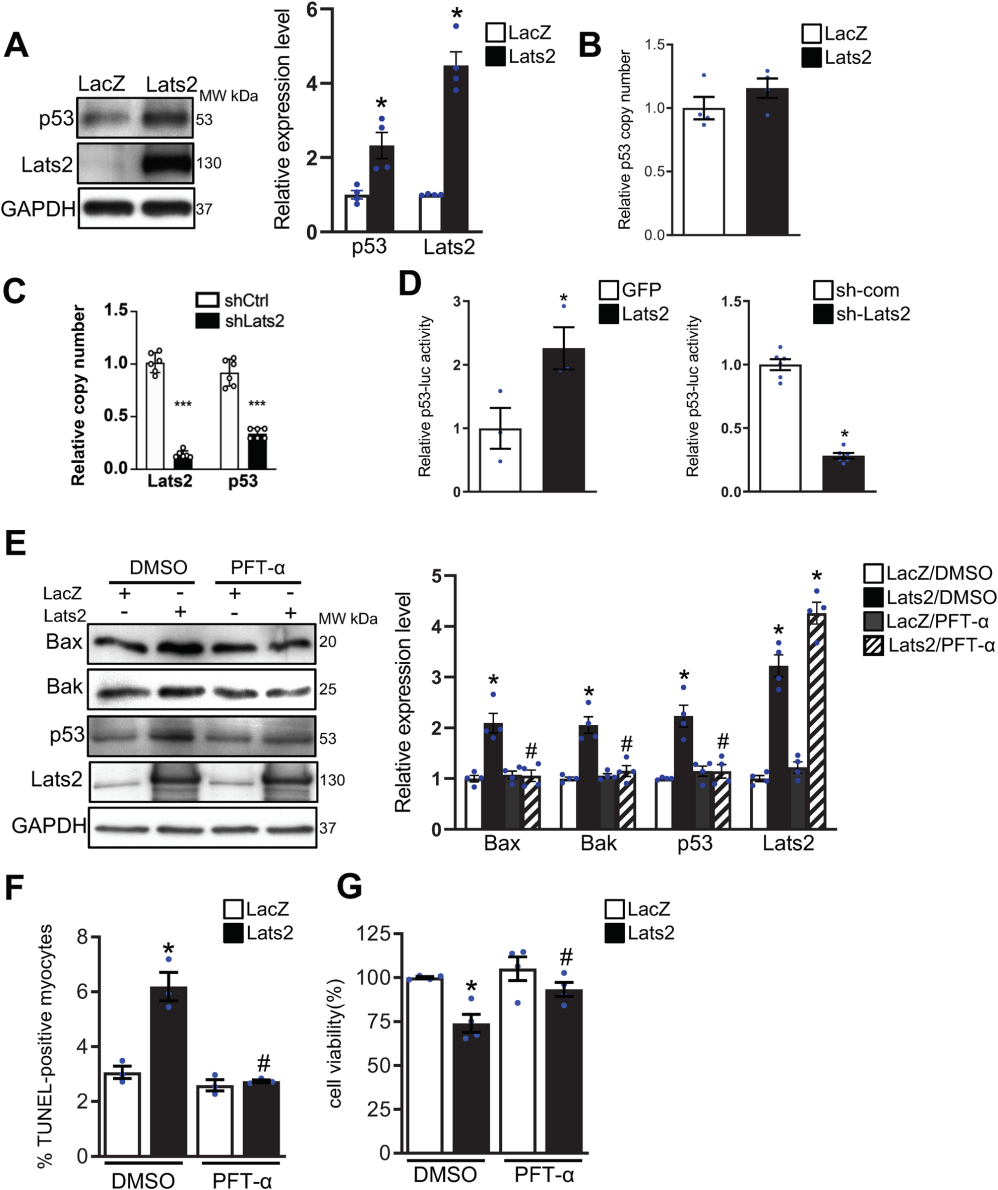
Lats2 upregulates Bax and Bak in a p53-dependent manner. (**A**) and (**B**), Cultured neonatal rat ventricular cardiomyocytes were transduced with adenovirus harboring either LacZ or Lats2. (**A**), Protein levels of p53, Lats2 and GAPDH were evaluated with immunoblot analyses. (**B**), mRNA levels of p53 were evaluated with qPCR analyses. (**C**), Cultured neonatal rat ventricular cardiomyocytes were transduced with adenovirus harboring either shRNA Lats2 or shRNA control. mRNA levels of p53 were evaluated with qPCR analyses. ***p < 0.001 versus Adenovirus harboring shRNA control. (n = 6). (**D**), Cultured neonatal rat ventricular cardiomyocytes were transfected with p53-luciferase reporter together with expression plasmids harboring either GFP (control) or Lats2 (left) and either shRNA-control or shRNA-Lats2. Relative luciferase activity is shown. The level of reporter gene activity in control (GFP or shRNA control) transfected cells was designated as 1. (**E**–**G**), Cultured neonatal rat ventricular cardiomyocytes were transduced with adenovirus harboring LacZ or Lats2 in the presence of Pifithrin-α (PFT-α), a p53 inhibitor, or dimethylsulfoxide (DMSO), a vehicle. (**E**), The levels of Bax, Bak, p53, Lats2 and GAPDH in cardiomyocytes were evaluated with immunoblot analyses. Representative images of immunoblot analyses are shown. Experiments were repeated three times. (**F**), Cardiomyocyte apoptosis was evaluated with TUNEL assays. (**G**), Cell viability was evaluated with CellTiter-Blue assays. In (**B**) and (**C**), *p < 0.05 versus LacZ-transduced cardiomyocytes in the presence of DMSO. #*p* < 0.05 versus Lats2-transduced cardiomyocytes in the presence of DMSO.

Lats2 inhibits the activity of Mdm2 through direct interaction and stabilizes p53 in cancer cells^17^. Interestingly, Adenovirus-mediated overexpression of Lats2 increased the protein level of p53 without affecting mRNA levels in cultured cardiomyocytes (Fig. 6A,B). Thus, Lats2 increases p53 through a posttranscriptional mechanism in a cell-autonomous manner in cardiomyocytes. Conversely, shRNA-mediated downregulation of Lats2 significantly decreased p53 protein and mRNA expression in cardiomyocytes (Fig. 6C). Overexpression of Lats2 increased whereas downregulation of Lats2 decreased, the activity of a p53-luciferase reporter gene in cultured cardiomyocytes, suggesting that Lats2 positively regulates the transcriptional activity of p53 in cardiomyocytes (Fig. 6D). In order to evaluate the role of p53 in mediating upregulation of Bax and Bak and Lats2-induced death^9^, we tested the effect of Pifithirin-α (PFT-α), a specific p53 inhibitor^18^. PFT-α inhibited Lats2-induced upregulation of Bax and Bak in cultured cardiomyocytes (Fig. 6E), suggesting that p53 plays an important role in mediating Lats2-induced upregulation of Bax and Bak in cardiomyocytes. Lats2-induced increases in apoptosis (Fig. 6F) and decreases in cell survival (Fig. 6G) in cultured cardiomyocytes were also abolished in the presence of PFT-α, suggesting that p53 plays an important role in mediating Lats2-induced cell death in cardiomyocytes.

## Discussion

Our results suggest that endogenous Lats2 plays a detrimental role during pressure overload in the postnatal heart. Lats2 is activated by TAC whereas haploinsufficiency of Lats2 attenuates pathological hypertrophy and LV dysfunction during pressure overload. The detrimental role of Lats2 may be mediated by a mechanism involving p53-mediated upregulation of Bax and Bak, proapoptotic Bcl-2 family proteins.

Lats2 induces apoptosis and inhibits hypertrophy in cultured cardiomyocytes in a cell autonomous manner^9^. We found that haploinsufficiency of Lats2 attenuates both cardiac dysfunction and hypertrophy in response to pressure overload. The alleviation of cardiac dysfunction in response to TAC appears to be explained by the inhibition of apoptosis caused by haploinsufficiency of Lats2. However, the effect of Lats2 haploinsufficiency upon hypertrophy appears to be overridden by the decrease in hemodynamic overload due to the improved cardiac function in Lats2 +/- mice in vivo. Thus, endogenous Lats2 may be more influential upon apoptosis than cell size in cardiomyocytes during TAC.

It should be noted, however, that cardiac specific overexpression of *dominant negative* Lats2 enhanced cardiac hypertrophy and inhibited cardiomyocyte apoptosis in response to TAC^9^. Although the effect upon cardiomyocyte apoptosis was reproduced here, we did not observe the enhancement of cardiac hypertrophy in Lats2 +/- mice in response to TAC. The difference may be due to the fact that systemic haploinsufficiency of Lats2 affects not only cardiomyocytes but also non-cardiomyocytes or that DN-Lats2 can inhibit both Lats1 and Lats2. It has been shown that loss of Lats2 function does not affect the level of Lats1 in liver-specific KO mice in vivo^19^. Since we and others have shown that the roles of the Hippo pathway components are cell-type dependent, it is likely that the lack of enhancement of cardiac hypertrophy in Lats2 +/- mice in response to TAC is attributable to the effect of Lats2 downregulation in non-myocytes. Further investigation is needed to clarify this issue. It should be noted that our study is translational in that systemic haploinsufficiency should mimic the conditions of inhibitor treatment in the clinical setting. Whereas germline mutation might elicit the development of various compensatory mechanisms, the overall effects of systemic and partial suppression of Lats2 achieved in Lats2 +/- mice should be more similar to the effects of inhibitor treatment. We speculate that systemic and 50% inhibition of Lats2 during pressure overload should delay the development of heart failure. This warrants further investigation to evaluate the effect of Lats2 suppression upon heart failure progression, using large animal models.

TAC-induced increases in LV dysfunction and heart failure are alleviated in Lats2 +/- mice, accompanied by decreases in apoptosis and fibrosis. We here show that Lats2 promotes apoptosis of cardiomyocytes through a p53-dependent mechanism, including Bax and Bak. A previous study showed that Lats2 and p53 stimulate each other’s activity in fibroblasts^17^. Thus, although Lats2 could affect other unknown signaling mechanisms in cardiomyocytes, we propose that p53-dependent activation of proapoptotic Bcl-2 family proteins and apoptosis contributes to the detrimental effects of Lats2 during pressure overload.

Lats2 directly phosphorylates YAP at Ser127, thereby inducing nuclear exit of YAP^16^. YAP nuclear localization and activity are regulated not only by the Hippo kinases, including Lats1 and Lats2, but also through Hippo kinase-independent mechanisms^3^. We have shown previously that YAP is activated acutely by TAC through RhoA-dependent mechanisms^11^ and that YAP is inactivated during the chronic phase of pressure overload^13^. The latter is accompanied by increases in YAP phosphorylation at Ser127. Here, we show that Lats2 is activated by pressure overload and that YAP phosphorylation at Ser127 is attenuated in Lats2 +/- mice. Thus, our results suggest that Lats2 could be an endogenous YAP kinase during the chronic phase of pressure overload.

We have shown previously that haploinsufficiency of YAP in mice promotes LV dysfunction during pressure overload^11^. Thus, it is likely that alleviation of YAP phosphorylation and inactivation in Lats2 +/- contributes to the improvement of LV function. The effect of Lats2 upon p53 is mediated independently of YAP^20^. However, we have shown previously that YAP promotes cardiomyocyte survival through FoxO1-induced upregulation of catalase and MnSOD^10^. Thus, how changes in YAP activity contribute to the salutary effects in Lats2 +/- mice during TAC remains to be elucidated.

In summary, systemic heterozygous downregulation of Lats2 significantly alleviates pathological hypertrophy during pressure overload in part by inhibiting apoptosis in cardiomyocytes. Thus, interventions to inhibit Lats2 may be useful for delaying the development of heart failure in patients with pressure overload. Further investigation regarding the underlying mechanism for the cardioprotection afforded by Lats2 inhibition may allow the development of effective and selective interventions to treat heart failure.

## Methods

### Animal experiments

All genetically modified mouse lines used in this study have been described previously^21,22^. The systemic Lats2 +/- mice were a generous gift from Dr. Dae Sik Lim (Korea). Systemic Lats2 KO mice are embryonic lethal, whereas Lats2 +/- mice were born in a Mendelian ratio and their phenotype was apparently normal at 5 months of age. Mice were housed in a temperature-controlled environment within a range of 21–23 °C with a 12-h light/dark cycle and were given free access to water and a standard diet. To collect heart samples, mice were euthanized by cervical dislocation. The numbers of animals used are shown in figure legends. Interventions were performed in a blind fashion. No animals were excluded from analyses. All procedures involving animals were performed in accordance with NIH guidelines and ARRIVE guidelines (http://www.nc3rs.org.uk/page.asp?id=1357) and protocols (PROTO9999000934 and PROTO201900140) approved by the ethics committee of Rutgers Biomedical and Health Sciences.

### Transverse aortic constriction (TAC)

The method for TAC has been described^13^. Mice (12–14 weeks old) were subcutaneously injected with a small volume of bupivacaine, anesthetized with pentobarbital (60–70 mg/kg, i.p.), and mechanically ventilated. Aortic constriction was performed by ligation of the transverse thoracic aorta between the innominate artery and left common carotid artery with a 27-gauge needle using a 7–0 braided polyester suture. After surgery, mice were allowed to recover in a Thermocare unit, followed by injection with Meloxicam-SR (4 mg/kg, s.c.).

### Echocardiography

Mice were anesthetized with 2.5% avertin (12 μL/g body weight, i.p.). Echocardiography was performed using ultrasonography (Acuson Sequoia C256, Siemens, USA)^13^. A 13 MHz linear ultrasound transducer was used. For all mouse experiments, data analysis was conducted in a blinded manner.

### Histological analysis

Heart specimens were fixed with 4% paraformaldehyde and sectioned at 6 μm thickness. Cardiomyocyte size was evaluated using wheat germ agglutinin (WGA) staining. Fibrosis was evaluated with picrosirius red staining. Quantification of cell size and fibrosis was conducted using wheat germ agglutinin (WGA)-conjugated CF488 (Biotium) staining and the ImageJ program. Cardiomyocyte apoptosis was evaluated with TUNEL staining as described previously^13^.

### Primary cultures of neonatal rat ventricular cardiomyocytes

Primary cultures of neonatal rat ventricular cardiomyocytes were prepared from approximately 100 pooled hearts of both male and female 1-day-old Hsd:WI Wistar rats (Envigo, USA)^13^. A cardiomyocyte-rich fraction was obtained by centrifugation through a discontinuous Percoll gradient. Cardiomyocytes were cultured in complete medium containing DMEM/F12 supplemented with 5% horse serum, 4 μg/mL transferrin, 0.7 ng/mL sodium selenite, 2 g/L bovine serum albumin, 3 mM pyruvate, 15 mM HEPES pH 7.1, 100 μM ascorbate, 100 mg/L ampicillin, 5 mg/L linoleic acid, and 100 μM 5-bromo-2’-deoxyuridine (Sigma) overnight. The medium was then changed to normal serum-free DMEM/F12 medium with penicillin/streptomycin. Culture dishes were coated with 0.3% gelatin. For treatment with adenoviruses, after changing the complete medium, Cardiomyocytes were transduced with the indicated adenoviruses in serum-free DMEM/F12 medium. The cell viability was evaluated with CellTiter-Blue (Promega) assays.

### Adenovirus construction

Recombinant adenovirus vectors for Lats2 overexpression were constructed, propagated, and tittered as previously described^12^. pBHGloxΔE1,3Cre plasmid was co-transfected with the pDC316 shuttle vector (Microbix) or pDCSilencer vector (Microbix) containing the gene of interest into HEK293 cells using Lipofectamine 2000 (Thermo Fisher).

### Immunoblotting

Cells and hearts were lysed with ice-cold lysis buffer composed of 10 mM Tris pH 7.5, 150 mM NaCl, 5 mM EDTA, 1% Triton-X, 50 mM NaF, and 10% glycerol containing protease inhibitor (Sigma-Aldrich) and 1 μM MG-132 (Sigma-Aldrich). The protein concentrations of samples were measured using a BCA protein assay kit (Thermo Fisher). The denatured protein samples (10–30 μg) were analyzed by Western blotting using the indicated antibodies. Signal intensities of bands were quantified with the ImageJ program.

### qPCR

Total RNA was prepared from cultured cardiomyocytes using TRIzol (Thermo Fisher). cDNA was generated using 1,000 ng total RNA and PrimeScript RT master mix (Takara). qPCR was performed using TB Green Premix Ex Taq (Takara). Ribosomal protein 18S was used as an internal control. The oligonucleotide primers used to carry out the qPCRs of 18S, Bax, Bak and p53 and 18S are listed below:

- 18S forward: AGGGGAGAGCGGGTAAGAGA.
- 18S reverse: GGACAGGACTAGGCGGAACA.
- Bax forward: GAGCGGCTGCTTGTCTGGAT.
- Bax reverse: CAAGGCAGCAGGAAGCCTCA.
- Bad forward: AATGGCATCCGGACAAGGAC.
- Bad reverse: TGTTCCTGCTGGTGGAGGTA.
- P53 forward: ACAGTCGGATATGAGCATCG.
- P53 reverse: CCATGGAATTAGGTGACCCT.
- GAPDH forward: CTCCCACTCTTCCACCTTCG.
- GAPDH reverse: GCCTCTCTTGCTCAGTGTCC.
- Bcl2 forward: ATGCCTTTGTGGAACTATATGGC.
- Bcl2 reverse: GGTATGCACCCAGAGTGATGC.
- Bcl-xL forward: AGGCGATGAGTTTGAACTGC.
- Bcl-xL reverse: TGAAGCTGGGATGTTAGATCACT.
- Lats2 forward: ATTCCTGGTGCGTGTCTGTTT.
- Lats2 reverse: CTGTTTGGGAGATGGGAGGG.

### Reporter gene assays

p53-luciferase reporter has been described^23^. Cells were plated on 12-well plates and transfected with 1 μg of the indicated luciferase vectors using Fugene 6. Total DNA quantity was kept constant. Six hours after transfection, the cells were washed and transduced with the indicated adenovirus. The cells were lysed with Passive Lysis Buffer, and the transcriptional activity was determined using a luciferase assay system (Promega).

### Antibodies

Antibodies against the following proteins were used: GAPDH (1:3000, 2118, Cell Signaling Technology (CST)), YAP (1:1000, 14,074, CST), pS127-YAP (1:1000, 4911, CST), Lats2 (1:1000, A300-479A-M, Bethyl Laboratories), Bcl-2 (1:1000, 3498, CST), Bcl-xL (1:1000, 2764, CST), Bax (1:1000, 14,796, CST), Bak (1:1000, 12,105, CST), Bad (1:1000, 9239, CST), p53 (1:2000, 2527, CST), and cardiac troponin T (1:400, MA5-12,960, Thermo Fisher). Anti-Lats2 Ser872 phospho-specific antibody and Anti-Lats2 Thr1041 phospho-specific antibody were obtained from Dr. Hiroshi Nojima’s laboratory (Osaka University).

### Statistical analysis

All values in graphs are expressed as the mean $\pm$ S.E. Normality was tested with the Shapiro–Wilk normality test. Two group comparisons were performed by unpaired t-test. Multiple group comparisons were performed by either one-way ANOVA followed by the Tukey post-test if the data exhibited a normal distribution or the Kruskal–Wallis test followed by Dunn’s post-test if a group failed normality testing. A priori power calculation was performed based on data from published studies^21,22,24^ and pilot experiments. The effect size in this study was 1–5 with an alpha = 0.05 and power = 0.80. Microsoft Excel 2016 was used for Student’s *t* tests and GraphPad Prism 8 was used for ANOVA and Kruskal–Wallis tests. All quantified Western blot and cell viability data are shown as values relative to control.

## Supplementary Information

Supplementary Information 1.

Supplementary Information 2.
