# Supplementary material for: Lats2 promotes heart failure by stimulating p53-mediated apoptosis during pressure overload
Source: Sci Rep. 2021 Dec 6;11:23469. doi: 10.1038/s41598-021-02846-3 (PMC8648781; doi:10.1038/s41598-021-02846-3)
Supplement: Supplementary file 1 — Supplementary Information 1. [file 41598_2021_2846_MOESM1_ESM.pdf]

Lats2 promotes heart failure by stimulating p53-mediated apoptosis during pressure overload

Dan Shao[1], Peiyong Zhai[1], Chengchen Hu[1], Risa Mukai[1], Sebastiano Sciarretta[2,3], Dominic Del Re[1], Junichi Sadoshima[1,4]

[1]Department of Cell Biology and Molecular Medicine, Rutgers New Jersey Medical School, USA

[2]Department of Medical and Surgical Sciences and Biotechnologies, Sapienza University of Rome, Latina, Italy

[3]IRCCS Neuromed, Pozzilli (IS), Italy

Running title: Lats2 promotes pathological hypertrophy

[4]Corresponding Author:
Junichi Sadoshima, MD, PhD
Department of Cell Biology and Molecular Medicine
Rutgers New Jersey Medical School
185 S Orange Ave, MSB G609
Newark, NJ 07103
E-mail: sadoshju@njms.rutgers.edu

Supplemental Figure S1

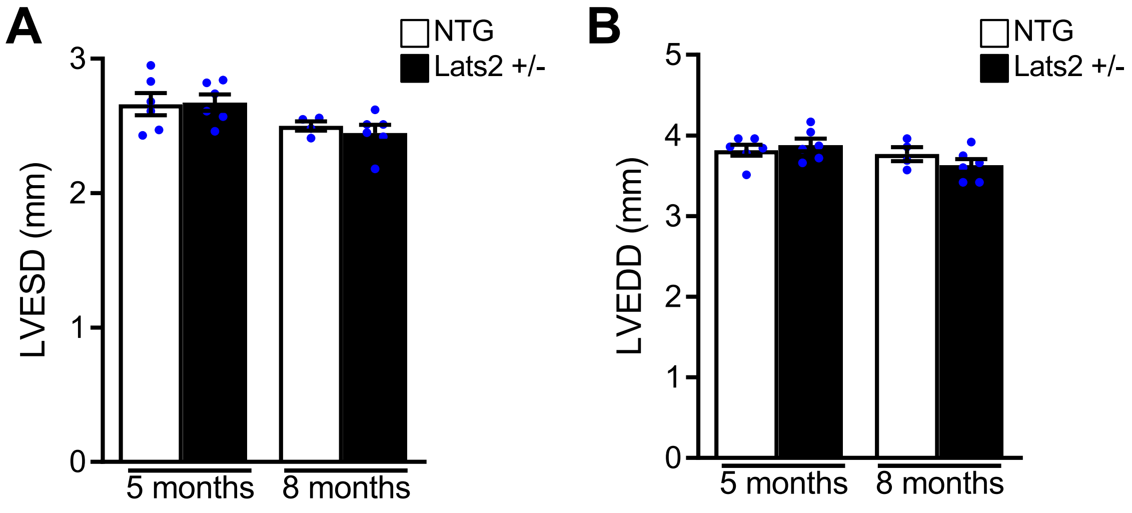

Supplemental Figure S1 Cardiac dimensions of Lats2+/- mice at 5 and 8 months of age at baseline. Non-transgenic (NTG) and Lats2+/- mice were euthanized at 5 or 8 months of age. A and B, Left ventricular end systolic dimension (LVESD) (A) and left ventricular end diastolic dimension (LVEDD) (B) were evaluated with echocardiographic analyses.

Supplemental Figure S2

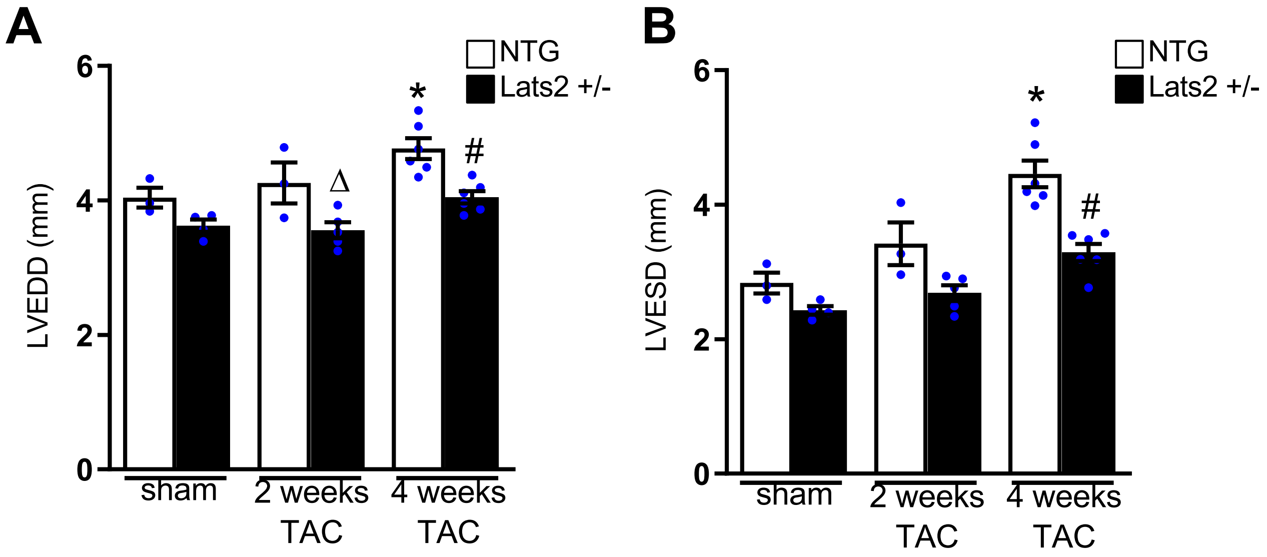

Supplemental Figure S2 Cardiac dimensions of Lats2+/- mice 2 and 4 weeks after TAC or sham operation. Non-transgenic (NTG) and Lats2+/- mice were subjected to either transverse aortic constriction (TAC) or sham operation for either 2 or 4 weeks. The results from the 2 and 4 weeks sham groups were combined. A and B, Left ventricular end systolic dimension (LVESD) (A) and left ventricular end diastolic dimension (LVEDD) (B) were evaluated with echocardiographic analyses. *p<0.05 vs. NTG mouse hearts with sham operation. #p<0.05 vs. NTG mouse hearts with TAC for 4 weeks. Δp<0.05 vs. NTG mouse hearts with TAC for 2 weeks.

Supplemental Table S1

	NTG	Lats2 +/-
n	8	10
BW(g)	23.88±1.34	23.05±2.08
TL(mm)	18.25±0.29	17.81±0.24
HW(mg)	107±9.93	106.75±5.44
LV(mg)	80.5±8.06	80.75±3.40
HW/BW(mg/g)	4.47±0.18	4.64±0.20
LV/BW(mg/g)	3.37±0.16	3.51±0.18
HW/TL(mg/mm)	5.86±0.47	5.99±0.23
LV/TL(mg/mm)	4.41±0.39	4.53±0.14
Lung(mg)	174±21.65	157±12.19
Liver(mg)	970.25±89.35	892.5±101.67
Lung/BW(mg/g)	7.33±1.23	6.82±0.16
Lung/TL(mg/mm)	9.54±1.22	8.81±0.57
Liver/BW(mg/g)	40.66±3.14	38.67±1.55
Liver/TL(mg/mm)	53.15±4.57	50.05±5.07

Supplemental Table S1 Baseline organ weights of Lats2+/- and control non-transgenic (NTG) mice at 5 months of age. BW body weight; TL tibial length; HW heart weight; LV left ventricular weight.

Supplemental Table S2

	NTG	Lats2 +/-
n	4	6
BW(g)	25.18±1.23	25.42±1.64
TL(mm)	17.75±0.5	17.75±0.42
HW(mg)	106±4.69	113.83±5.38*
LV(mg)	80±2.58	86.17±3.76*
HW/BW(mg/g)	4.21±0.064	4.48±0.13*#
LV/BW(mg/g)	3.18±0.053	3.40±0.13*
HW/TL(mg/mm)	5.98±0.37	6.41±0.25
LV/TL(mg/mm)	4.51±0.22	4.85±0.15*
Lung(mg)	160±13.66	166±17.18
Liver(mg)	1199±80.21	1136.5±66.91
Lung/BW(mg/g)	6.35±0.34	6.52±0.48
Lung/TL(mg/mm)	9.01±0.72	9.35±0.90
Liver/BW(mg/g)	47.63±2.35	44.74±1.15*
Liver/TL(mg/mm)	67.68±6.44	64.03±3.46

*p<0.05 vs NTG
#p<0.01 vs NTG

Supplemental Table S2 Baseline organ weights of Lats2+/- and control non-transgenic (NTG) mice at 8 months of age. BW body weight; TL tibial length; HW heart weight; LV left ventricular weight.

Supplemental Table S3

Age	5 months		8 months	
	NTG	Lats2+/-	NTG	Lats2+/-
n	6	6	4	6
DSEP WT (mm)	0.74±0.04	0.85±0.06	0.77±0.01	0.86±0.04
LVEDD(mm)	3.82±0.07	3.88±0.08	3.77±0.09	3.63±0.08
DPW WT(mm)	0.76±0.02	0.78±0.03	0.83±0.04	0.84±0.01
SSEP WT(mm)	1.23±0.02	1.28±0.07	1.12±0.03	1.34±0.05
LVESD(mm)	2.66±0.08	2.67±0.06	2.50±0.04	2.45±0.06
SPW WT(mm)	1.04±0.02	1.11±0.05	1.13±0.05	1.04±0.05*
LVEF(%)	66±2	67±2	71±2	69±2
%FS	30±2	31±1	34±2	33±1
HR(/min)	451±16	443±29	458±13	466±32

Supplemental Table S3 Baseline echocardiographic analyses of Lats2+/- and control non-transgenic (NTG) mice at 5 months and 8 months of age. DSEP WT, diastolic septal wall thickness; LVEDD Left ventricular end diastolic dimension; DPW WT diastolic posterior wall thickness; SSEPW WT systolic septal wall thickness; LVESD left ventricular end systolic wall thickness; LVEF left ventricular ejection fraction; %FS % fractional shortening; HR heart rate. *p<0.05 vs. NTG.

Supplemental Table S4

Age	5 months		8 months	
	NTG	Lats2+/-	NTG	Lats2+/-
n	6	7	3	6
SBP(mmHg)	87.33±3.00	86.86±3.57	88±9.80	91.67±4.91
DBP(mmHg)	60±3.86	52±3.70	57.3±8.16	60±5.27
MBP(mmHg)	69.11±3.27	63.62±3.62	67.56±8.71	70.56±5.11
LVSP(mmHg)	91.33±2.62	92±3.17	89.33±6.53	94±4.00
LVEDP(mmHg)	4.67±0.67	3±0.41	2.67±0.82	3.33±0.42
dP/dtmax(mmHg)	5917±539	6500±338	7167±1021	7667±749
dP/dt min(mmHg)	6000±465	7083±326	7000±816	7500±606
HR(/min)	463±11	484±23	482±32	508±11

Supplemental Table S4 Baseline hemodynamic analyses of Lats2+/- and control non-transgenic (NTG) mice at 5 months and 8 months of age. SBP systolic blood pressure; DBP diastolic blood pressure; MBP mean blood pressure; LVSP left ventricular systolic pressure; LVEDP left ventricular end diastolic pressure; HR heart rate.

Supplemental Table S5

	sham		4 weeks TAC	
	NTG	Lats2+/-	NTG	Lats2+/-
n	4	4	6	6
DSEP WT (mm)	0.83±0.09	0.96±0.05	1.47±0.10*	1.32±0.08*
LVEDD(mm)	4.04±0.15	3.62±0.09	4.77±0.16*	4.05±0.09#
DPW WT(mm)	0.70±0.05	0.7±0.03	0.92±0.04*	1.02±0.05*
SSEP WT(mm)	1.17±0.16	1.23±0.08	1.62±0.09	1.60±0.12
LVESD(mm)	2.84±0.16	2.43±0.06	4.46±0.20*	3.29±0.13#
SPW WT(mm)	0.93±0.12	1.06±0.04	0.94±0.03	1.27±0.06*#
LVEF(%)	65±2	69±3	19±4*	46±4*#
%FS	30±1	33±2	7±1*	19±2*#
HR(/min)	450±30	490±22	521±14	504±26

Supplemental Table S5 Echocardiographic analyses of Lats2+/- and control non-transgenic (NTG) mice after 4 weeks of transverse aortic constriction (TAC) or sham operation. DSEP WT, diastolic septal wall thickness; LVEDD Left ventricular end diastolic dimension; DPW WT diastolic posterior wall thickness; SSEPW WT systolic septal wall thickness; LVESD left ventricular end systolic wall thickness; LVEF left ventricular ejection fraction; %FS % fractional shortening; HR heart rate. *p<0.05 vs. sham. #p<0.05 vs. NTG.

Supplemental Table S6

	sham		4 weeks TAC	
	NTG	Lats2+/-	NTG	Lats2+/-
n	4	4	4	6
SBP(mmHg)	82±3.46	86.67±4.81	156.5±15.84	171.33±6.48
DBP(mmHg)	53±4.12	54.67±4.81	76±7.11	73.33±4.70
MBP(mmHg)	62.67±3.89	65.33±4.68	102.84±9.74	106±4.28
Gradient (mmHg)	N/A	N/A	81.5±13.40	96.67±7.62
LVSP(mmHg)	90±2	89.33±3.53	139±11.36	180.67±6.67[#]
LVEDP(mmHg)	2.5±0.5	2.67±0.67	10.5±1.89	9.33±0.84
dP/dtmax(mmHg)	7000±661	6667±481	6500±574	8133±698
dP/dt min(mmHg)	6800±400	6133±533	6300±526	8000±685
HR(/min)	453±8	456±12	486±32	489±29

Supplemental Table S6 Hemodynamic analyses of Lats2+/- and control non-transgenic (NTG) mice after 4 weeks of transverse aortic constriction (TAC) or sham operation. SBP systolic blood pressure; DBP diastolic blood pressure; MBP mean blood pressure; LVSP left ventricular systolic pressure; LVEDP left ventricular end diastolic pressure; HR heart rate.
